# Bevacizumab plus chemotherapy in nonsquamous non‐small cell lung cancer patients with malignant pleural effusion uncontrolled by tube drainage or pleurodesis: A phase II study North East Japan Study group trial NEJ013B

**DOI:** 10.1111/1759-7714.13472

**Published:** 2020-05-18

**Authors:** Rintaro Noro, Kunihiko Kobayashi, Jiro Usuki, Makiko Yomota, Masaru Nishitsuji, Tsuneo Shimokawa, Masahiro Ando, Mitsunori Hino, Koichi Hagiwara, Akihiko Miyanaga, Masahiro Seike, Kaoru Kubota, Akihiko Gemma, North East Japan Study group

**Affiliations:** ^1^ Department of Pulmonary Medicine and Oncology Graduate School of Medicine, Nippon Medical School Tokyo Japan; ^2^ Department of Respiratory medicine Saitama Medical University International Medical Center Saitama Japan; ^3^ Department of Pulmonary Medicine Nippon Medical School Musashikosugi Hospital Kawasaki Japan; ^4^ Department of Thoracic Oncology and Respiratory Medicine Tokyo Metropolitan Cancer and Infectious disease Center Komagome Hospital Tokyo Japan; ^5^ Department of Respiratory Medicine Ishikawa Prefectural Central Hospital Kanazawa Japan; ^6^ Department of Respiratory Medicine and Medical Oncology Yokohama Municipal Citizen's Hospital Yokohama Japan; ^7^ Department of Internal Medicine Jizankai Medical Foundation Tsuboi Cancer Center Hospital Fukushima Japan; ^8^ Department of Pulmonary Medicine Nippon Medical School Chiba Hokuso Hospital Chiba Japan; ^9^ Division of Pulmonary Medicine Jichi Medical University Tochigi Japan

**Keywords:** Bevacizumab plus chemotherapy, non‐small cell lung cancer, unsuccessful management of malignant pleural effusion, vascular endothelial growth factor

## Abstract

**Background:**

Pleurodesis is the standard of care for non‐small cell lung cancer (NSCLC) patients with symptomatic malignant pleural effusion (MPE). However, there is no standard management for MPE uncontrolled by pleurodesis. Most patients with unsuccessful MPE control are unable to receive effective chemotherapy. Vascular endothelial growth factor (VEGF) plays an important role in the pathogenesis of MPE. This multicenter, phase II study investigated the effects of bevacizumab plus chemotherapy in nonsquamous NSCLC patients with unsuccessful management of MPE.

**Methods:**

Nonsquamous NSCLC patients with MPE following unsuccessful tube drainage or pleurodesis received bevacizumab (15 mg/kg) plus chemotherapy every three weeks. The primary endpoint was pleural effusion control rate (PECR), defined as the percentage of patients without reaccumulation of MPE at eight weeks. Secondary endpoints included pleural progression‐free survival (PPFS), safety, and quality of life (QoL).

**Results:**

A total of 20 patients (median age: 69 years; 14 males; 20 adenocarcinomas; six epidermal growth factor receptor mutations) were enrolled in nine centers. The PECR was 80% and the primary end point was met. The PPFS and the overall survival (OS) were 16.6 months and 19.6 months, respectively. Patients with high levels of VEGF in the MPE had shorter PPFS (*P* = 0.010) and OS (*P* = 0.002). Toxicities of grade ≥ 3 included neutropenia (50%), thrombocytopenia (10%), proteinuria (10%), and hypertension (2%). The cognitive QoL score improved after treatment.

**Conclusions:**

Bevacizumab plus chemotherapy is highly effective with acceptable toxicities in nonsquamous NSCLC patients with uncontrolled MPE, and should be considered as a standard therapy in this setting.

**Key points:**

**Significant findings of the study:**

Bevacizumab plus chemotherapy is highly effective with acceptable toxicities in nonsquamous NSCLC patients with uncontrolled MPE.

**What this study adds:**

Bevacizumab plus chemotherapy should be considered as a standard treatment option for patients with uncontrolled MPE.

**Clinical trial registration:**

UMIN000006868 was a phase II study of efficacy of bevacizumab plus chemotherapy for the management of malignant pleural effusion (MPE) in nonsquamous non‐small cell lung cancer patients with MPE unsuccessfully controlled by tube drainage or pleurodesis (North East Japan Study Group Trial NEJ‐013B) (http://umin.sc.jp/ctr/).

## Introduction

Malignant pleural effusion (MPE) is a common complication in non‐small cell lung cancer (NSCLC). Uncontrolled MPE may cause chest distress, shortness of breath, and breathing difficulty. This leads to a deterioration in patient quality of life (QoL) and prevents patients from receiving effective chemotherapy.^1^, ^2^, ^3^ Furthermore, MPE is associated with poor prognosis and has been classified as stage IV disease, according to the eighth edition of the Tumor‐Node‐Metastasis classification for lung cancer.^4^, ^5^, ^6^ The standard management of symptomatic massive MPE is intercostal drainage followed by pleurodesis using talc and other agents, including OK‐432.^3^, ^7^, ^8^

Prospective and retrospective studies on intrapleural therapy of MPE have reported that the success rate of controlling pleural effusion at 2.5 months was only 50%–70%.^9^, ^10^, ^11^, ^12^, ^13^, ^14^, ^15^ In cases of unsuccessful pleurodesis or insufficient expansion of the lung after drainage, most patients with NSCLC are unable to receive effective chemotherapy.

Vascular endothelial growth factor (VEGF) is recognized as a key molecule for the formation and pathogenesis of MPE.^16^, ^17^, ^18^ The humanized, anti‐VEGF, monoclonal antibody bevacizumab inhibits the proliferation, migration, and differentiation of vascular endothelial cells. In addition, bevacizumab promotes apoptosis of endothelial cells and suppresses VEGF‐induced neoangiogenesis and vascular permeability. Of note, bevacizumab has been shown to synergize with chemotherapeutic agents to avert the accumulation of pleural fluid.^19^, ^20^, ^21^, ^22^

However, these clinical studies, including a phase II study of platinum‐based chemotherapy combined with bevacizumab, such as NEJ013A trial, have indicated promising efficacy in patients with limited and controllable MPE without drainage or pleurodesis.^19^, ^20^, ^21^, ^22^

Currently, there is no established standard therapy for the management of MPE in nonsquamous NSCLC patients with MPE unsuccessfully controlled by tube drainage or pleurodesis. Thus, the present multicenter, phase II study was conducted to evaluate the effect of bevacizumab plus chemotherapy in nonsquamous NSCLC patients with unsuccessful management of MPE.

## Methods

### Patient selection

Eligible patients included those aged ≥20 years, with histologically or cytologically confirmed stage IV nonsquamous NSCLC and recurrence after resection, along with MPE unsuccessfully controlled by tube drainage or pleurodesis. Other eligibility criteria included an Eastern Cooperative Oncology Group (ECOG) performance status (PS) of 0–2, and an estimated life expectancy of ≥12 weeks. Laboratory requirements were as follows: hemoglobin level of ≥9.0 g/dL, white blood cell count of ≥3000/mm^3^, absolute neutrophil count of ≥1500/mm^3^, platelet count of ≥100 000/mm^3^, serum bilirubin level of ≤1.5 mg/dL, aspartate aminotransferase and alanine aminotransferase levels of ≤100 IU/L, creatinine clearance of ≥45 mL/min, and proteinuria ≤1+. An SpO2 level of ≥90% was also required.

This multicenter, phase II study was conducted in centers in Japan. The study protocol was approved by the institutional review boards of the participating centers, and the study was conducted in accordance with the principles of the Declaration of Helsinki and the Guidelines for Good Clinical Practices. All patients provided written informed consent prior to the performance of the study‐specific procedures. This study was registered in the University Hospital Medical Information Network in Japan (UMIN) Clinical Trials Registry with number: UMIN000006868.

### Treatment schedule

Nonsquamous NSCLC patients with MPE, who had previously received unsuccessful tube drainage or pleurodesis with insufficient expansion of the lung, received at least two cycles of bevacizumab (15 mg/kg) plus chemotherapy every three or four weeks. After completion of four cycles of bevacizumab plus chemotherapy, maintenance chemotherapy plus bevacizumab was administered every three or four weeks until disease progression. In case of recurrence due to reasons other than reaccumulation of MPE, an alternative regimen chosen by the treating physician was administered until reaccumulation of MPE.

In case of an increase in MPE after initiation of treatment, the treatment was discontinued. In cases of proteinuria ≥2+, subsequent treatment with bevacizumab was withheld until proteinuria returned to ≤1+.

### Study assessments

Assessments included pleural effusion control rate (PECR) (primary endpoint) and pleural progression‐free survival (PPFS) without reaccumulation of MPE, treatment‐related adverse events, and evaluation of QoL using the European Organization for Research and Treatment for Cancer Quality of Life Questionnaire‐Core 30 (EORTC QLQ‐C30) (secondary endpoints). Reaccumulation was defined as an unequivocal increase in MPE compared with baseline observed through chest radiography or computed tomography. PECR was defined as the percentage of patients without re‐accumulation of MPE eight weeks after initiation of treatment. PPFS without reaccumulation of MPE was calculated from the time of treatment initiation until the day pleural effusion increased again regardless of other aspects of disease progression, or the day of death regardless of cause. Overall survival (OS) was defined as the time from enrollment in the study until death regardless of cause. During the study, physical examination, weight, vital signs, ECOG PS, complete blood count, blood chemistry, and urinalysis were conducted/monitored on day 1 of each treatment cycle. For the assessment of adverse events, the National Cancer Institute Common Terminology Criteria for Adverse Events grading system was used (CTCAE, version 4.0). The Kaplan‐Meier method was employed to estimate PPFS and OS.

VEGF levels in the plasma and MPE were measured at baseline. The samples were stored at −80°C until measurement. Levels of VEGF in the plasma and MPE were measured using an enzyme immunoassay.^20^ The cutoff values for low and high levels of VEGF in the plasma were defined according to the median level (VEGF <260.6 pg/mL and VEGF ≥260.6 pg/mL, respectively). Similarly, low and high levels of VEGF in the MPE were defined according to the median level (VEGF <9874.8 pg/mL and VEGF ≥9874.8 pg/mL, respectively). The QoL was evaluated using the established EORTC QLQ‐C30.^23^ Patients completed this questionnaire at baseline (prior to initiation of bevacizumab‐based therapy) and eight weeks after treatment initiation.

### Statistical analysis

The one‐arm binomial method (SWOG statistical tools) was used to determine the study sample size. Assuming that an MPE control rate of 40% would indicate potential usefulness of the treatment, whereas an MPE control rate of 10% would indicate lack of efficacy with α = 0.05 and β = 0.10, it was calculated that 17 patients were required for the present study. The investigators planned to recruit 20 patients to compensate for possible study discontinuations.

Survival analysis of PPFS and OS were performed using the Kaplan–Meier method. Patients without disease progression at the data cutoff point (November 2016) were censored as being progression‐free. Differences in survival time between subgroups were analyzed using the log‐rank test. All statistical analyses were performed using the GraphPad Prism 7 (GraphPad Software, Inc., La Jolla, CA, USA) and SPSS 25 (IBM SPSS Statistics, Inc., Chicago, IL, USA) software. A *P*‐value <0.05 denoted statistical significance.

## Results

### Patient characteristics

A total of 20 patients were enrolled in the study from August 2012 to June 2016. The characteristics of these patients are shown in Table 1. The median age was 69 years (range: 52–73 years) and the majority (14/20 patients; 70%) were males. All patients had stage IV adenocarcinoma. Six patients (30%) had epidermal growth factor receptor (EGFR) mutations. Prior to enrolment, the initial management of MPE consisted of 12 pleurodeses: OK‐432 in 10 cases and talc in two cases, and only tube drainage in eight cases without sufficient expansion of the lung.

**Table 1:** Patient characteristics

			*N* = 20 (%)
Age (range)			69 (52–73)
Gender		Male	14 (70%)
		Female	6 (30%)
PS		0	8 (40%)
		1	12 (60%)
		2	0 (0%)
Smoking status		Never	5 (25%)
		Ex/Current	15 (75%)
Clinical stage of lung cancer		Stage IV	20 (100%)
		Recurrence	0 (0%)
Histology		Adenocarcinoma	20 (100%)
Status of *EGFR* mutation		Negative	14 (70%)
		Positive	6 (30%)
Status of ALK gene rearrangement		Negative	20 (100%)
		Positive	0 (0%)
Regimen of combined chemotherapy		CBDCA+PTX	6 (30%)
		CBDCA+PEM	10 (50%)
		others	4 (20%)
The state of MPE with unsuccessful tube drainage or pleurodesis		Full lung expansion following tube drainage and adherent therapy	12 (60%)
		Without re‐expansion following tube drainage	8 (40%)

ALK, anaplastic lymphoma kinase; CBDCA, carboplatin; EGFR, epidermal growth factor receptor; MPE, malignant pleural effusion; PEM, pemetrexed; PS, performance status; PTX, paclitaxel.

The duration between the administration of bevacizumab and removal of the chest tube after drainage and pleurodesis was 17.85 days (4–76 days). The sufficient duration would not affect delay wound healing by bevacizumab.

The combination regimen of chemotherapy plus bevacizumab (15 mg/kg) consisted of carboplatin‐pemetrexed (10 patients), carboplatin‐paclitaxel (six patients), docetaxel (two patients), pemetrexed (one patient), and erlotinib (one patient). Six patients with *EGFR* mutation received combination therapy with bevacizumab targeting uncontrolled MPE after treatment using EGFR tyrosine kinase inhibitors.

Chemotherapy cycles were repeated every three or four weeks and patients received a median of eight cycles (range: 1–20) of combination chemotherapy. There were 13 patients where treatment was discontinued due to disease progression while treatment was discontinued in four patients due to adverse events (pulmonary thrombosis [*n* = 1], renal function [*n* = 1], arrhythmia [*n* = 1], and cerebral infarction [*n* = 1]).

### Efficacy

**Figure 1:**
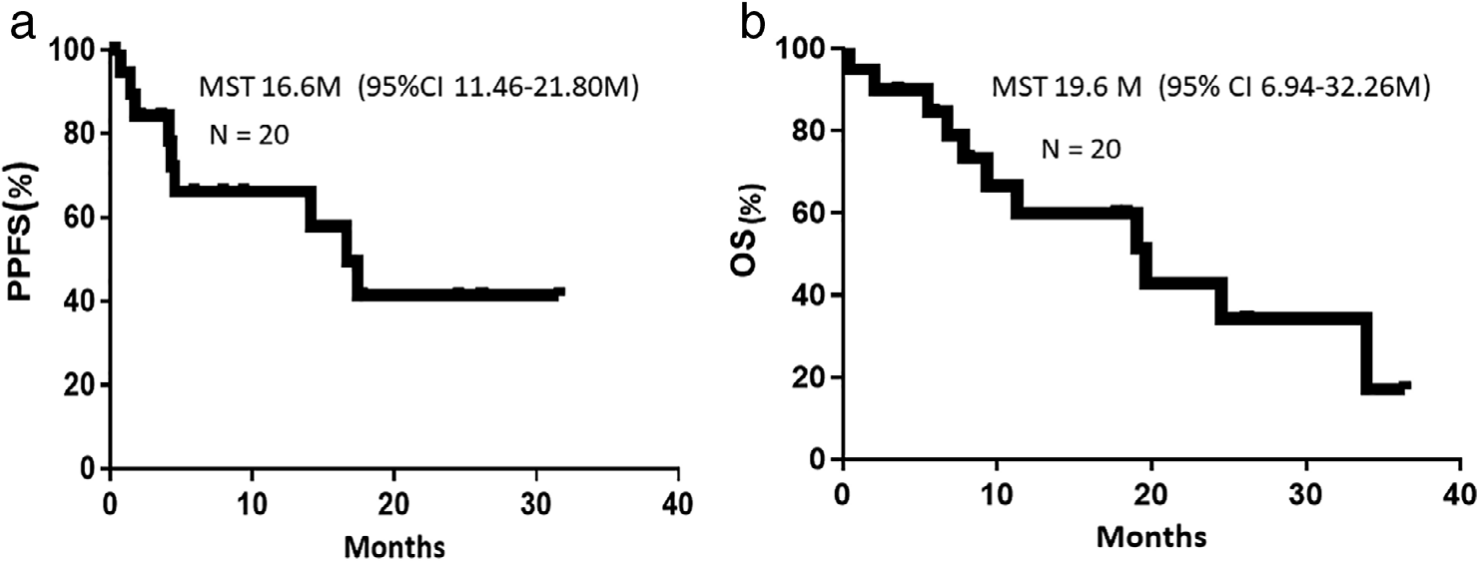
(**a**) Pleural progression‐free survival (PPFS). (**b**) Overall survival in the study.

The control rate of MPE without pleurodesis at eight weeks was 80.0% (95% confidence interval [CI]: 78.0–82.0) and the primary endpoint was met (Table 2). Three of eight patients showed reaccumulation without re‐expansion following tube drainage. Only one of 12 patients showed reaccumulation within eight weeks after full‐lung expansion following tube drainage and pleurodesis. There was no significant difference in PECR between patients with and without full lung expansion (Fisher’s exact test: *P* = 0.255). Median pleurodesis‐free survival was 16.6 months (Fig 1a). The final survival assessment was carried out in November 2016. Median OS was 19.6 months (95% CI: 6.9–32.3 months) (Fig 1b).

**Table 2:** Control rate of malignant pleural effusion (MPE) without deterioration of pleurodesis eight weeks after initiation of treatment

	*N* = 20 (%)
Without reaccumulation of MPE, eight weeks after initiation of treatment	16 (80%)
With reaccumulation of MPE, eight weeks after initiation of treatment	4 (20%)

### Safety

Table 3 shows the incidence of adverse events in this study. The incidence of grade ≥ 3 adverse events was as follows: neutropenia (50%), leukopenia (20%), thrombocytopenia (10%), proteinuria (10%), hypertension (10%), pulmonary embolism (5%), central nervous system ischemia (5%), arrhythmia (5%), and infection (5%). Febrile neutropenia or treatment‐related deaths were not observed in this study.

**Table 3:** Adverse events

Hematological	All grades	%	Grade ≥ 3	%
Neutropenia	13	65	10	50
Leukopenia	11	55	4	20
Thrombocytopenia	8	40	2	10
Anemia	10	50	0	0
**Nonhematological**	**All Grades**	**%**	**Grade ≥ 3**	**%**
Proteinuria	10	50	2	10
Hypertension	10	50	2	10
Pulmonary embolism	1	5	1	5
Infection	1	5	1	5
CNS ischemia	1	5	1	5
Supraventricular and nodal arrythmia	1	5	1	5
Vomiting	1	5	0	0
BUN	1	5	0	0
Fever	1	5	0	0
Edema	1	5	0	0
Insomnia	1	5	0	0
Chills	1	5	0	0
Hyperglycemia	1	5	0	0
Hiccups	1	5	0	0
Tinnitus	1	5	0	0
Na	1	5	0	0
LDH	1	5	0	0
Alopecia	2	10	0	0
Alb	2	10	0	0
Dyspnea	2	10	0	0
Arthritis (nonseptic)	2	10	0	0
Cre	2	10	0	0
Nausea	3	15	0	0
Eruption	3	15	0	0
Peripheral neuropathy: Motor	3	15	0	0
Stomatitis	3	15	0	0
Diarrhea	4	20	0	0
Constipation	5	25	0	0
Peripheral neuropathy: Sensory	5	25	0	0
Loss of appetite	7	35	0	0
Fatigue	8	40	0	0
GOT/GPT increased	10	50	0	0

Alb, albumin; CNS, central nervous system; Cre, creatinine; GOT, glutamate oxalacetate transaminase; GPT, glutamate pyruvate transaminase; LDH, lactate dehydrogenase; Na, sodium.

### Association of VEGF levels with clinical outcomes

**Figure 2:**
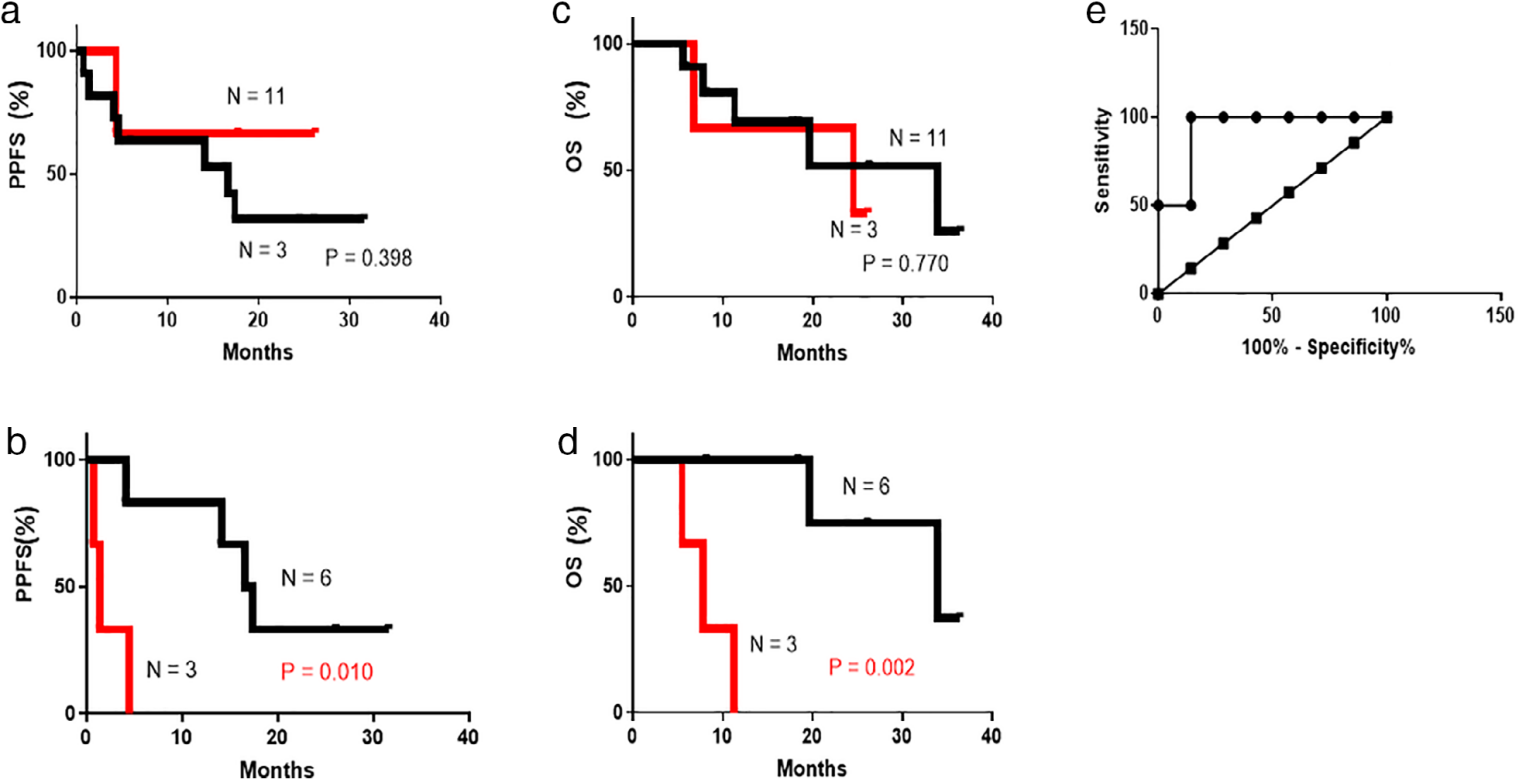
Pleural progression‐free survival (PPFS) and overall survival (OS) according to VEGF levels in the plasma and MPE. The cutoff value was estimated according to the median levels of VEGF. (**a**) PPFS according to the levels of VEGF in the plasma at baseline (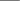) VEGF low in plasma, (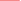) VEGF high in plasma. (**b**) PPFS according to the levels of VEGF in MPE at baseline (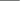) VEGF Low in MPE, (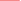) VEGF High in MPE. (**c**) OS according to the levels of VEGF in the plasma at baseline (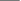) VEGF low in plasma, (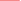) VEGF high in plasma. (**d**) OS according to the levels of VEGF in the MPE at baseline (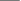) VEGF Low in MPE, (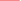) VEGF high in MPE. (**e**) Cutoff value of the levels of VEGF in MPE at baseline obtained from the ROC curve (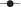) Sensitivity%, (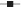) Identity%. VEGF, vascular endothelial growth factor; MPE, malignant pleural effusion; ROC, receiver operating characteristic.

The levels of VEGF in the plasma and MPE were measured prior to the initiation of chemotherapy in 14 and nine patients, respectively. The level of VEGF (median ± standard deviation) in the plasma and MPE at baseline was 260.6 ± 391.8 pg/mL and 9875.0 ± 12 208.0 pg/mL, respectively. On the other hand, receiver operating characteristic (ROC) curve analyses based on PECR, provided a cutoff value of 9140 pg/ml (area under the curve, AUC 0.929, sensitivity: 100% [95% CI: 15.81–100%]; 85.71% [95% CI: 42.13% to 99.64%]). There was no significant extramural relationship between the levels of VEGF in the plasma and MPE prior to the initiation of chemotherapy in nine patients (r = −0.6, *P* = 0.1). The level of VEGF in the plasma was not associated with PPFS (high levels of VEGF: 24.5 months; low levels of VEGF: 14.1 months; log‐rank test: *P* = 0.398). However, PPFS was shorter in patients with higher levels of VEGF in the MPE than those with lower levels of VEGF, using a cutoff based on the median value(9875 pg/mL) or ROC curve analyses (9140 pg/mL) (high levels of VEGF: 1.2 months; low levels of VEGF: 17.2 months; log‐rank test: *P* = 0.010) (Fig 2a–e).

The median OS of patients with higher levels of VEGF in the MPE was significantly shorter than in patients with lower levels of VEGF (log‐rank test: *P* = 0.002) (Fig 2d).

### Quality of life (QoL)

**Figure 3:**
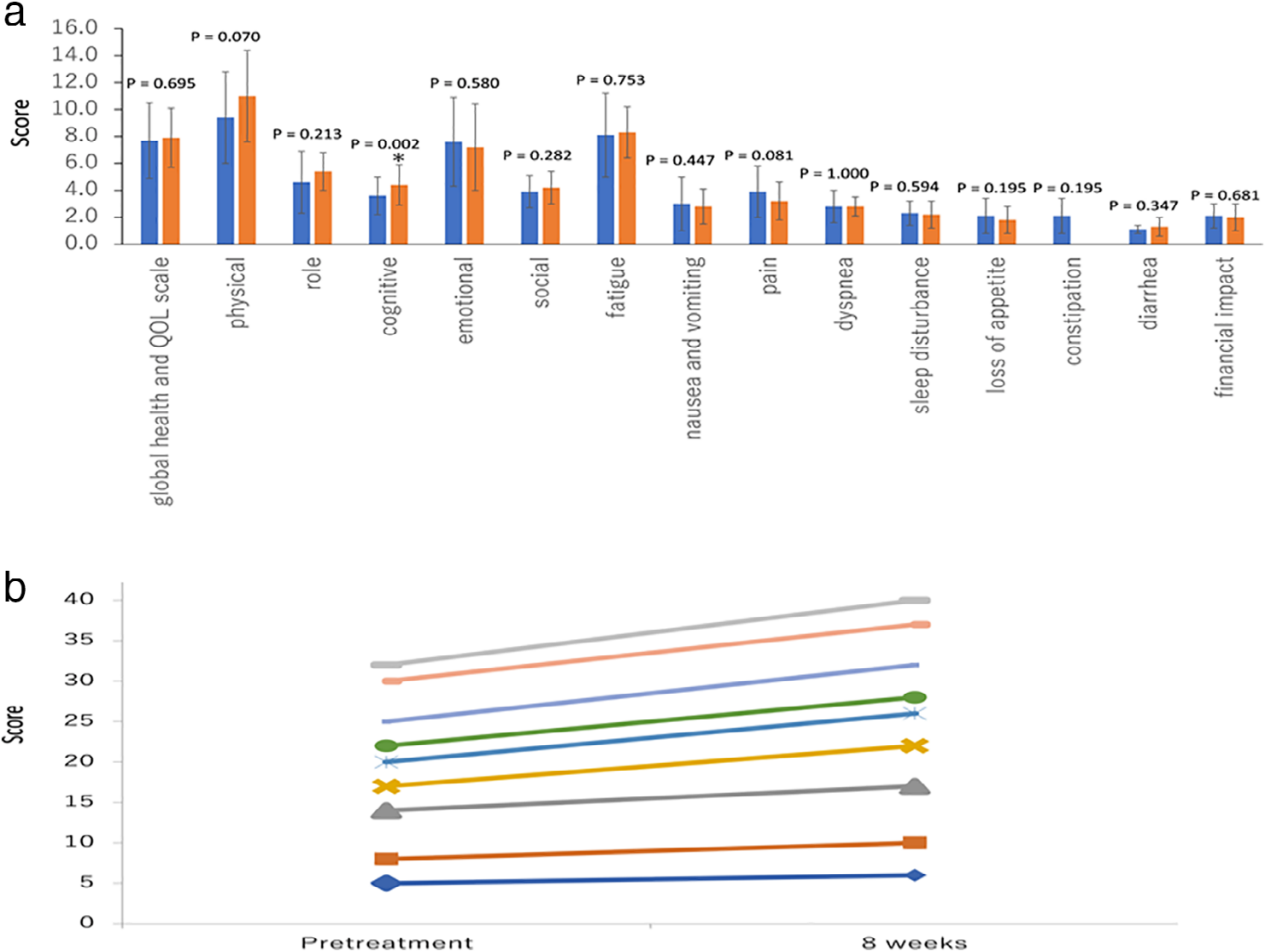
Assessment of quality of life (QoL) using the EORTC QLQ‐C30. (**a**) Improvement between before treatment and after eight weeks using a paired *t*‐test. The analysis of QoL, evaluating physical functioning and pain eight weeks after initiation of bevacizumab‐based therapy, showed a trend towards improvement compared with baseline (*P* = 0.070 and 0.081, respectively). (**b**) Improvement in cognitive scoring for nine patients (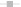) Case 9, (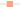) Case 8, (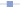) Case 7, (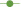) Case 6, (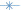) Case 5, (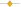) Case 4, (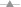) Case 3, (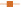) Case 2, (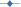) Case 1. QoL, quality of life; EORTC QLQ‐C30, European Organization for Research and Treatment for Cancer Quality of Life Questionnaire‐Core 30.

Nine patients completed the questionnaires using the established EORTC QLQ‐C30 questionnaire developed to assess the QoL of patients with cancer. The results showed that cognitive QoL, evaluated eight weeks after initiation of bevacizumab plus chemotherapy, improved compared with baseline (*P* = 0.002) (Fig 3a,b). Similarly, QoL in terms of physical functioning and pain showed a trend towards improvement eight weeks after initiation of treatment compared with baseline (*P* = 0.070 and 0.081, respectively) (Fig 3a).

## Discussion

Prospective and retrospective studies on intrapleural therapy of MPE, which was recognized as a standard management, have reported that the success rate of controlling pleural effusion at 2.5 months was only 50%–70%.

Unsuccessful control of MPE worsens the prognosis for patients with NSCLC and additional management for uncontrolled MPE is required.^24^, ^25^ Some clinical studies, including a phase II study of platinum‐based chemotherapy combined with bevacizumab such as NEJ013A trial, have indicated promising efficacy in patients with limited and controllable MPE without repeated drainage or pleurodesis.^19^, ^20^, ^21^, ^22^ The present multicenter, phase II study evaluated the effects of bevacizumab‐containing therapy in nonsquamous NSCLC patients with unsuccessful management of MPE. Using this treatment, PECR was 80% (95% CI: 78.0–82.0), and the primary endpoint was met. At a median follow‐up period of 17.7 months, median PPFS and median OS were 16.6 and 19.6 months, respectively. Cognitive QoL had improved eight weeks after initiation of bevacizumab plus chemotherapy compared with baseline (*P* = 0.002).

VEGF plays an important role in the pathogenesis of MPE.^16^, ^17^, ^18^, ^26^, ^27^, ^28^, ^29^ Malignant cancer cells invade or metastasize the pleural cavity and induce a VEGF‐related mechanism. VEGF is a potent growth factor for endothelial cells, inducing the formation of new blood vessels and accelerating vascular permeability (50000‐fold more potent than histamine). Malignant exudates showed universal induction activity regardless of tumor type and origin, and that noncancer stem cells underwent epithelial–mesenchymal transition and progressed into a cancer stem cell state de novo when exposed to MPEs/ascites. This process involves VEGF mediating the activation of the PI3K/mTOR pathway.^27^ Thus, VEGF is thought to induce effusion and accumulation.^16^, ^26^, ^27^, ^28^, ^29^

Bevacizumab is a humanized, anti‐VEGF, monoclonal antibody with demonstrated antitumor properties in lung cancer cell lines and animal models.^30^ The results from in vitro studies have demonstrated that this monoclonal antibody is able to effectively neutralize and inhibit almost all VEGF‐mediated activities.^31^, ^32^

According to the previous experimental studies, the anti‐VEGF antibody significantly and synergistically reduced the accumulation and pleural thickness of pleural fluid after tube drainage and pleurodesis in animals.^33^, ^34^, ^35^, ^36^ These experimental results may support our clinical benefit for combination therapy with bevacizumab after pleurodesis.

Levels of VEGF are high in effusions from various malignancies, such as mesotheliomas, as well as breast and lung cancers.^18^ Increased levels of VEGF play an important role in the development and accumulation of MPE. However, chemotherapy with bevacizumab prevented the effects of elevated VEGF levels and controlled the MPE. Currently, there are no established biomarkers and surrogate markers predicting the efficacy of bevacizumab. The patients with high levels of VEGF in the MPE had shorter PPFS (*P* = 0.010) and OS (*P* = 0.002), and our results supported the findings of Tamiya *et al*.^22^ Partially, the levels of VEGF in the MPE may be a surrogate marker for predicting successful control of previously uncontrollable MPE after pleurodesis. A limitation of this study is the small sample size. Furthermore, investigation is warranted to confirm the value of VEGF levels as a surrogate marker.

The toxicity of the treatment in the present study was tolerable. There were no severe or unique adverse events observed in the present study. Finally, we estimated the outcome in terms of the QOL. Eight weeks after initiation of bevacizumab‐based therapy, cognitive function was improved compared with baseline (*P* = 0.002). In addition, physical functioning and measurement of pain showed a trend towards improvement compared with baseline (*P* = 0.070 and 0.081, respectively). The control of pleural effusion and reduction in pleural invasion may contribute to the improvement of physical functioning and pain. Consequently, the improvement of physical functioning, pain, and chest tightness may contribute to the improvement of cognitive function by increasing multiple mental abilities, including learning, thinking, reasoning, memory, and decision, in the absence of brain metastasis. These QoL factors are important for the evaluation of MPE control. This was the first report not only QoL but PPFS and OS, were improved after bevacizumab‐based therapy for uncontrollable MPE.

In conclusion, as no standard therapy has been established for the management of MPE in nonsquamous NSCLC patients with MPE unsuccessfully controlled by tube drainage or pleurodesis, bevacizumab plus chemotherapy should be considered as a standard treatment option for patients with uncontrollable MPE.

## Disclosure

Dr Gemma, Dr Hagiwara, Dr Yomota and Dr Kobayashi, reported personal fees from Chugai, and Dr Kubota and Dr Seike reported grants and personal fees from Chugai. There is no conflict for other coauthors.
